# Pure Medicines

**Published:** 1852-09

**Authors:** 


### UNIVERSITY OF LOUISVILLE.

### PURE MEDICINES.

In a recent number of this journal, we noticed the fact that the Faculty of the Medical Department of the University of Louisville had sent out very liberal orders to Paris, and elsewhere, for preparations designed to illustrate the teachings of the several chairs of Anatomy, Physiology, Surgery, and Obstetrics, and that very valuable and extensive additions had already been made to the cabinet of Materia Medica, from the establishment of Philip Schieffelen, Haines & Co., Druggists and Chemists, New York. We omitted, at the time, to notice as fully as we desired, the great excellence of all the articles furnished by this house, for we really think that the zeal and enterprise which these gentlemen have manifested, in regard to the proper preparation and purity of medicines, are worthy of special notice, and special commendation.

The practice of medicine depends for successful results, upon the harmonious co-operation of a variety of circumstances, and when this harmony is disturbed, it is liable to prove more injurious than beneficial. Directed by principles deemed correct, as far as scientific inquiry and experience have been able to ascertain, and plentifully supplied with the implements most essential, and approved in its prosecution, it still often falls short of its beneficent purposes, and encounters mortification and defeat. The reasonable mind views a certain amount of failure with composure, for it appreciates the fallibility of human power, and knows that limits are placed, by the laws of nature, to the successful exertion of its efforts; but when the causes of failure are such as need not, and should not exist, the most lenient disposition feels constrained to condemn.

Among the many causes of the failure of curative medicine, there is one which we hope ere long to see entirely obviated by the efforts of such men as Philip Schieffelen, Haines & Co., and others who have given themseves earnestly and in good faith, to the work; we allude to the sale and use of imperfect and adulterated medicines. Very few of the medical profession, and fewer still of the general public had any adequate conception, some years ago, of the extent to which the manufacture and sale of spurious and adulterated medicines was carried, in this country and elsewhere; some startling developments made by a few curious and reliable inquirers into the matter led to the enactment, by Congress, of an inspection law, which, in its searching execution, has unfolded a most monstrous and appalling condition of things in reference to the traffic in medicines, and at the same time, has done very much to ameliorate this condition. Much, however, yet remains to be done; much that can only be done by the exercise of professional vigilance, and the proper direction of professional patronage. This vigilance should look to the character and responsibility of every house concerned in the preparation, purchase and sale of medicines, and this patronage should be extended only to such as can produce unequivocal testimonials of their claims to confidence. There are a great many persons engaged in vending medicins who are wholly destitute of the necessary preparatory education, and who have embarked in the business, simply because they have considered it a profitable investment of capital, trusting for success to shrewd dealing, large profits, chance, and the ignorance of their customers. Many succeed, and succeed very handsomely, and by their success, lure others no better than themselves into the business; but their success is purchased at the public expense, and by the infliction of incalculable damage upon all who fall within the range of their influence. This should not, and would not be if medical men, sustained by public authority, would determine to prevent it, by drawing a broad and distinct line between the competent, and educated, and progressive apothecary, who engages in the business in a scientific way, and the mere speculator, who has taken it up as a profitable trade. In the former of these classes we have reason to think that Philip Schieffelen, Haines & Co., deserve to be ranked, and therefore, have thought proper to notice them somewhat emphatically, disclaiming, at the same time, any special or exclusive partiality for them, beyond that which we feel for all who can present equal claims to the confidence of the profession.

